# Adsorption of doripenem and meropenem antibiotics on activated carbon derived from snake fruit seeds: single-compound and binary mechanism via experiments and modelling

**DOI:** 10.1038/s41598-026-41972-8

**Published:** 2026-03-11

**Authors:** Elham A. Alzahrani, Lotfi Sellaoui, Felycia Edi Soetaredjo, Mohamed Bouzidi, Abdalla Abdelwahab, Nuha Othman S. Alsaif, Nawal S. Alshammari, Alessandro Erto, Suryadi Ismadji

**Affiliations:** 1 Department of Chemistry, College of Science, University of Ha’il, 81451 Ha’il, Saudi Arabia; 2 CRMN, Centre for Research On Microelectronics and Nanotechnology of Sousse, NANOMISENE (LR16CRMN01), 4054 Sousse, Tunisia; 3 Laboratory of Quantum and Statistical Physics, Faculty of Sciences of Monastir, LR18ES18, Monastir University, Monastir, Tunisia; 4 Department of Chemical Engineering, Widya Mandala Surabaya Catholic University, Kalijudan 37, Surabaya, 60114 Indonesia; 5 Department of Physics, College of Science, University of Ha’il, P.O. Box 2440, Ha’il, Saudi Arabia; 6 Dipartimento di Ingegneria Chimica, dei Materiali e della Produzione Industriale, Università di Napoli Federico II, P.leTecchio, 80, 80125 Napoli, Italy

**Keywords:** Activated carbon, Emerging contaminants, Doripenem, Meropenem, Adsorption modeling, Antibiotic removal, Chemistry, Environmental sciences

## Abstract

The increasing presence of pharmaceutical contaminants in aquatic environments raises serious concerns regarding water quality and public health. In this study, activated carbon derived from snake fruit seeds was developed and applied for the adsorption of two β-lactam antibiotics, doripenem (DOR) and meropenem (MER), in both single and binary aqueous systems. The adsorbent was characterized using scanning electron microscopy (SEM), X-ray diffraction (XRD), and nitrogen adsorption–desorption isotherms. The results confirmed the development of a rough and porous surface morphology, and a mesoporous network with high surface area (1260.61 m^2^/g), which are favorable for adsorption. Based on single experimental data, the maximum adsorption capacities are193 mg/g for DOR and 171 mg/g for MER. These performances were reduced when a second adsorbate (MER or DOR) is present in solution, reflecting a competition on the same adsorbent site. The model for binary solutions contains number of molecules per site (n_1_, n_2_) explaining the antagonist effect between both adsorbates when interacting for the same activated carbon receptor sites (ACRS). Comparatively, it was demonstrated that our adsorbent is an efficient material for various pharmaceuticals removal. In summary, the activated carbon indicated promising performance to remove various pharmaceuticals including MER and DOR.

## Introduction

Aquatic contamination by pharmaceutical complexes has been considered as a serious environmental subject because of its damaging effects on ecosystems as well as human health^1^. In recent decades, the worldwide demand for pharmaceuticals has amplified substantially, driven by the significant increase of health issues and the continuous progress of new drugs^2^. This tendency has inexorably resulted in the release of pharmaceutical compounds into the environment, especially through wastewater discharges. In order to ensure high-quality and safe water, the treatment of wastewater has therefore become a central topic of research. Several treatment technologies have been suggested to moderate this issue and to prevent the discharge of polluted wastes into cleaner ecosystems^3,4^.

In this regard, adsorption technique has attracted noticeable attention as a robust, and cost-effective method for water purification. This technique is characterized by its simplicity in manipulation and its excellent removal performance at low pollutant concentrations. It has been regarded as one of the advantageous methods to remove various types of water pollutants. Indeed, a wide range of water pollutants is present in the aquatic systems, leading to serious environmental and health problems. Comparatively, adsorption offers better performance for molecules that are intractable, resistant to biodegradation, or present in trace amounts than conventional treatment techniques (e.g., chlorination, coagulation–flocculation, or biological degradation). The abovementioned advantages strongly justify the extensive application of adsorption in the removal of pharmaceuticals from aquatic media, where achieving effective pollutant removal is often challenging using traditional methods. Among these adsorption-based approaches, activated carbon is one of the most effective adsorbents owing to its exceptional characteristic such as its tunable porosity, its high surface area, and its strong affinity toward a large variety of pollutants^5–7^. Several works have established the excellent performance of activated carbon in removing various groups of pollutants, including dyes, pesticides, pharmaceuticals, and heavy metals^8–11^.

Among the various agricultural wastes that have been utilized as activated carbon precursors, the potential of some region-specific biomasses remains largely unexplored. Salak seeds (Salacca zalacca), also known as Snake fruit, are an abundant agro-industrial waste in Southeast Asia and have a high fixed carbon content, yet they have never been exploited to produce adsorbent materials. In this study, snake fruit seeds were converted into activated carbon through CO₂–KOH double activation, resulting in a large surface area and more developed mesoporosity compared to most reported fruit waste-based activated carbons. These characteristics provide advantages for the adsorption of large antibiotic molecules. To date, there are no reports of the use of snake fruit seed-based activated carbon to remove carbapenem antibiotics such as doripenem (DOR) and meropenem (MER), either in a single or multicomponent system.

In addition to its chemical characteristics, which make it suitable as an activated carbon precursor, the use of snake fruit seeds also has important sustainability value. Snake fruit seeds are a highly abundant agro-industrial waste in Indonesia and Southeast Asia, with national snake fruit production reaching hundreds of thousands of tons per year. Most of the seeds have no economic value and are typically discarded as organic residue. Therefore, their use as adsorbent raw material does not compete with food needs and does not require procurement costs. The use of this waste reduces the production cost of activated carbon. Moreover, it offers eco-friendly advantages by decreasing the volume of organic waste and increasing the added value of wasted biomass. Furthermore, the use of these seeds could improve regional and national economy by supporting the principles of circular economy. Thus, the selection of snake fruit seeds as an activated carbon precursor is based on its material features, availability, cost, and its sustainability aspects required for high efficiency pollutant removal.

For a detailed analysis of the adsorption mechanism of pharmaceuticals, diverse models can be applied on experimental data including Langmuir, Freundlich, Temkin, and Redlich–Peterson, in single and binary solutions. In this direction, Minaei et al.^12^ investigated the adsorption of sulfamethoxazole and lincomycin on H₃PO₄-modified activated sludge-based biochar using different models to ascribe insights into competitive adsorption phenomena. Similarly, Dhiman^13^ applied Langmuir and Freundlich models to study the adsorption of ciprofloxacin hydrochloride and ofloxacin hydrochloride onto Oryza sativa husk ash, highlighting differences between non-competitive and competitive adsorption in single and binary systems. Amin Zamiri et al.^14^ also investigated the adsorption of sulfamethoxazole and trimethoprim on biomass-based activated carbon using Langmuir and Nitta models. These mentioned models provided classical explanations of single and binary data. However, the adsorption mechanisms were not completely interpreted because they have certain limitations in their formulations. For a better understanding of the single and binary adsorption mechanisms, the application of advanced models derived from statistical physics represent an excellent option to avoid classical investigations^15–19^.

Both DOR and MER belong to the carbapenem class of β-lactam antibiotics, which are considered last-resort drugs for the treatment of multidrug-resistant bacterial infections. Their presence in the aquatic environment is of particular concern, as even trace concentrations can contribute to the development and dissemination of antimicrobial resistance, a major global health threat. Wastewater containing carbapenems, if untreated, poses risks not only to aquatic organisms but also to public health due to the potential horizontal transfer of resistance genes. This makes the study of their removal from water bodies especially critical.

Therefore, the main objective of the present study is to apply enhanced statistical physics-based adsorption models to analyze single-compound and binary adsorption data of DOR and MER onto activated carbon derived from snake fruit seeds. Specifically, the study aims to (i) provide molecular-level insights into the adsorption mechanisms of carbapenem antibiotics, (ii) assess the role of competitive interactions in binary systems, and (iii) evaluate the potential of low-cost, biomass-derived activated carbon for advanced water treatment applications.

## Experimental section

### Materials

The snake fruit (*Salaccazalacca*) seeds used in this study were obtained from a local market in Surabaya, Indonesia, and were of the Pondoh variety. Industrial-grade nitrogen gas (99.9% purity), employed to prevent oxidation during thermal processing, and carbon dioxide gas (99.9% purity), used as the activating agent, were supplied by PT Aneka Gas, Surabaya, Indonesia. Doripenem (C₁₅H₂₄N₄O₆S₂) and meropenem (C₁₇H₂₅N₃O₅S), both β-lactam antibiotics, were purchased from a local pharmaceutical distributor (Surabaya, Indonesia). All chemicals and reagents were of analytical grade and were used without further purification.

### Activated carbon preparation

Snake fruit seeds represent a lignocellulosic biomass waste with considerable potential as a precursor for activated carbon, thanks to their relatively high carbon content and structural framework, which can ensure adequate pore development. The proximate analysis of the seeds used in this study indicated a fixed carbon content of 34.4%, volatile matter of 48.1%, moisture content of 13.2%, and the remainder consisting of ash.

Moisture, volatile matter, ash, and fixed carbon content in snake fruit seeds were determined by proximate analysis according to standard biomass characterization methods. Briefly, moisture content was determined by drying the sample at 105 ± 2 °C to a constant mass. Volatile matter was measured by heating the dried sample in a closed crucible at 950 ± 20 °C for 7 min. Ash content was obtained by thermal oxidation at 750 ± 25 °C to a constant mass, while fixed carbon was calculated based on the difference:

$${\text{FC }}\left( \% \right)\, = \,{1}00{-}\left( {{\mathrm{moisture}}\, + \,{\text{volatile matter}}\, + \,{\mathrm{ash}}} \right).$$

Activated carbon (AC) was prepared from snake fruit seeds by employing a combined activation strategy, integrating physical activation with carbon dioxide (CO₂) and chemical activation with potassium hydroxide (KOH). This combined strategy is followed to optimize pore development and surface area.

In the first step, the seeds were oven-dried to remove residual moisture, crushed, and sieved to obtain a particle size fraction of 40/60 mesh. The carbonization process was carried out in a tubular furnace at 500 °C for 2 h under a continuous nitrogen flow (6 L/min) to prevent oxidation. Following carbonization, the resulting char was subjected to physical activation by exposure to CO₂ gas at 850 °C for 1 h, with the nitrogen flow replaced by CO₂ at 3 L/min.

The physically activated carbon was subsequently impregnated with an aqueous KOH solution at a mass ratio of 2:1 (carbon: KOH) and stirred for 3 h. This chemical activation step was designed to further develop the pore structure and generate additional micropores through chemical reactions between KOH and carbon. The impregnated material was then dried and thermally reactivated at 600 °C for 1 h under a nitrogen atmosphere.

The resulting AC was washed with diluted hydrochloric acid (HCl) to remove residual KOH and potassium salts, followed by repeated rinsing with deionized water until the filtrate reached neutral pH. Finally, the sample was oven-dried at 105 °C until a constant weight was obtained.

### Characterization of activated carbon

Characterization of AC from snake fruit seeds was carried out to evaluate the specific surface area (BET), surface morphology (SEM), and crystal structure (XRD).

### Adsorption experiments for single-compound and binary systems

Adsorption isotherm studies were conducted by varying the adsorbent mass between 0.1 and 1.0 g for each experimental setup. Stock solutions of DOR and MER were prepared at an initial concentration of 250 mg/L, and 100 mL aliquots were transferred into sealed Erlenmeyer flasks. The adsorption experiments were performed at three different temperatures (30 °C, 40 °C, and 50 °C)to evaluate the effect of temperature on adsorption capacity.

Each system was agitated for 3 h in an orbital shaker at a constant speed, corresponding to the equilibrium time determined from preliminary kinetic studies. At the end of the contact period, the suspensions were filtered, and the residual concentrations of DOR and MER in the filtrates were quantified using high-performance liquid chromatography (HPLC).

Binary adsorption experiments were carried out under identical conditions to those of the single-solute systems. For these experiments, equimolar solutions were prepared containing 250 mg/L of DOR and 250 mg/L of MER in 100 mL of solution. The binary adsorption tests were likewise conducted at 30 °C, 40 °C, and 50 °C.

During all adsorption experiments, the solution pH was not externally adjusted and was kept at the natural pH of the working DOR and MER solutions. This operational pH was close to neutrality, which is relevant to practical water treatment environments and ensures that no chemical modification was introduced to alter the intrinsic speciation of the antibiotics.

## Single-compound and competitive adsorption models

The analysis of experimental data in both single-compound and binary systems was carried out by employing two different models derived from statistical physics and expressing physical mechanisms compatible with the observed experimental data, reported in the following section.

The single-compound systems were modeled on the assumption that a relationship is established between the adsorbate and an active site, to which a defined density and adsorption energy is associated.

The binary system was modelled assuming that the coexistence of DOR and MER led to a reduction in the overall adsorption performance of the activated carbon (AC) adsorbent, indicating a competitive adsorption effect between the two compounds. The description of this phenomenology was carried out by two different theoretical models that were applied to fit the experimental data set at different temperatures.

### Model for single-compoundsystems

According to this model, the adsorption of DOR and MER on activated carbon (AC) occurs through the formation of an adsorbed layer on the AC surface. The adsorption mechanism of both compounds is governed by an adsorption energy parameter that characterizes the interactions between the adsorbates and the AC surface. Unlike the classical Langmuir model, this approach allows each adsorption site to accommodate a variable number of molecules. The model expression is given as^20–22^:

1 $${Q}_{e}=\frac{n{D}_{m}}{1+{\left(\frac{{C}_{hs}}{C}\right)}^{n}}$$

where *n* represents the number of DOR or MER molecules that can be adsorbed per activated carbon receptor site (ACRS), C_hs_ denotes the concentration at half-saturation (i.e., when half of the ACRSs are occupied), and D_m_ corresponds to the density of ACRSs.

### Model for binary system

This model hypothesizes a reduction in the adsorption capacities of DOR and MER, primarily attributed to competitive adsorption on the same activated carbon receptor sites (ACRS). This model therefore assumes that each ACRS can accommodate either DOR or MER molecules. Accordingly, two parameters, denoted as n_1_ and n_2_, are introduced to represent the number of DOR and MER molecules adsorbed per active site on adsorbent surface, respectively. In addition, two binary adsorption energy terms are incorporated into the model to describe the interactions of DOR and MER with the ACRSs. The mathematical expression of the model is given as^23,24^:

2 $${Q}_{a1}=\frac{{n}_{1}{N}_{M}(\frac{{c}_{1}}{{c}_{01}}{)}^{{n}_{1}}}{1+(\frac{{c}_{1}}{{c}_{01}}{)}^{{n}_{1}}+(\frac{{c}_{2}}{{c}_{02}}{)}^{{n}_{2}}}$$

3 $${Q}_{a2}=\frac{{n}_{2}{N}_{M}(\frac{{c}_{2}}{{c}_{02}}{)}^{{n}_{2}}}{1+(\frac{{c}_{1}}{{c}_{01}}{)}^{{n}_{1}}+(\frac{{c}_{2}}{{c}_{02}}{)}^{{n}_{2}}}$$

Both equations express the variation of the adsorption capacities of DOR and MER in the binary system, respectively, and include several defined parameters: *n*_*1*_ and *n*_*2*_ represent the number of DOR and MER molecules adsorbed per activated carbon receptor site (ACRS), while *C*_*01*_ and *C*_*02*_ correspond to the concentrations at half-saturation for DOR and MER, respectively. When binary saturation is reached, the density of the receptor sites is represented by the parameter N_m_.

## Results and discussion

### Adsorbent characterization

**Fig. 1:**
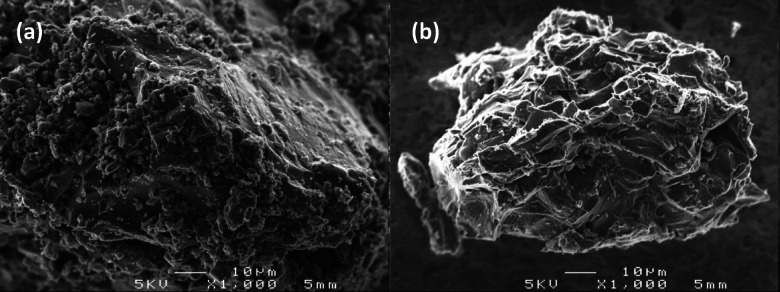
SEM images of the AC derived from snake fruit seeds: (**a**) before adsorption, and (**b**) after adsorption of the antibiotics doripenem and meropenem.

Figure 1 shows a scanning electron microscope (SEM) image of the activated carbon (AC) derived from snake fruit seeds at 1000 × magnification. Image (a) shows the AC surface before adsorption, while image (b) shows the activated carbon surface after adsorption of the antibiotics DOR and MER.

In Fig. 1a, the surface morphology of the AC appears rough and heterogeneous, with small particles distributed across the matrix surface. Such morphology indicates the existence of several open cavities and pores, confirming therefore, the effectiveness of the combined physical and chemical activation processes. It should be noted that the formation of such porous structure is a main indicator of adsorption efficacy. Indeed, this structure offers a large surface area as well as abundant active sites accessible for interaction with adsorbate molecules.

Figure 1b displays the surface morphology of the AC after adsorption of DOR and MER. The surface looks smoother, and numerous pores are observed to be partially covered by a relatively homogeneous layer. This observation suggests that the antibiotic molecules were successfully adsorbed and partially blocked the active sites of the carbon surface. This result designates that the adsorption process was effective, involving possible physical and chemical interactions between the functional groups of the activated carbon and the active moieties of DOR and MER. Overall, the most evident morphological changes are pore narrowing and surface smoothing, which directly confirm the deposition of antibiotic molecules on the activated carbon surface.

X-ray diffraction (XRD) measurement was carried out to examine the crystallinity and phase structure of the AC sample. For AC materials, XRD typically shows broad peaks around 2θ = 22–26° associating with to the (002) plane of graphite, and weaker peaks around 2θ = 43–45° corresponding to the (100) plane. The aforementioned diffraction peaks are characteristic of a disordered graphitic structure. In this study, direct XRD analysis of DOR and MER was not feasible, as both antibiotics were obtained in injectable form. Therefore, reference peak positions were taken from the literature and patent data. The characteristic diffraction peaks of DOR molecules are reported at 2θ ≈ 10.95, 13.12, 15.03, 15.95, 16.64, 18.14, 18.99, 19.71, 20.66, 21.12, 22.22, 23.40, 23.96, 26.13, 27.05, 27.51, 28.29, 29.02, 31.73, and 33.45° (U.S. Patent No. US9840506B2). For MER, characteristic peaks are found at 2θ ≈ 8.62, 9.76, 12.17, 12.56, 12.99, 15.23, 16.20, 17.20, 18.33, 19.79, 20.24, 21.34, 22.03, 23.69, 24.54, 25.19, and 26.31° (European Patent EP2938617B1).

**Fig. 2:**
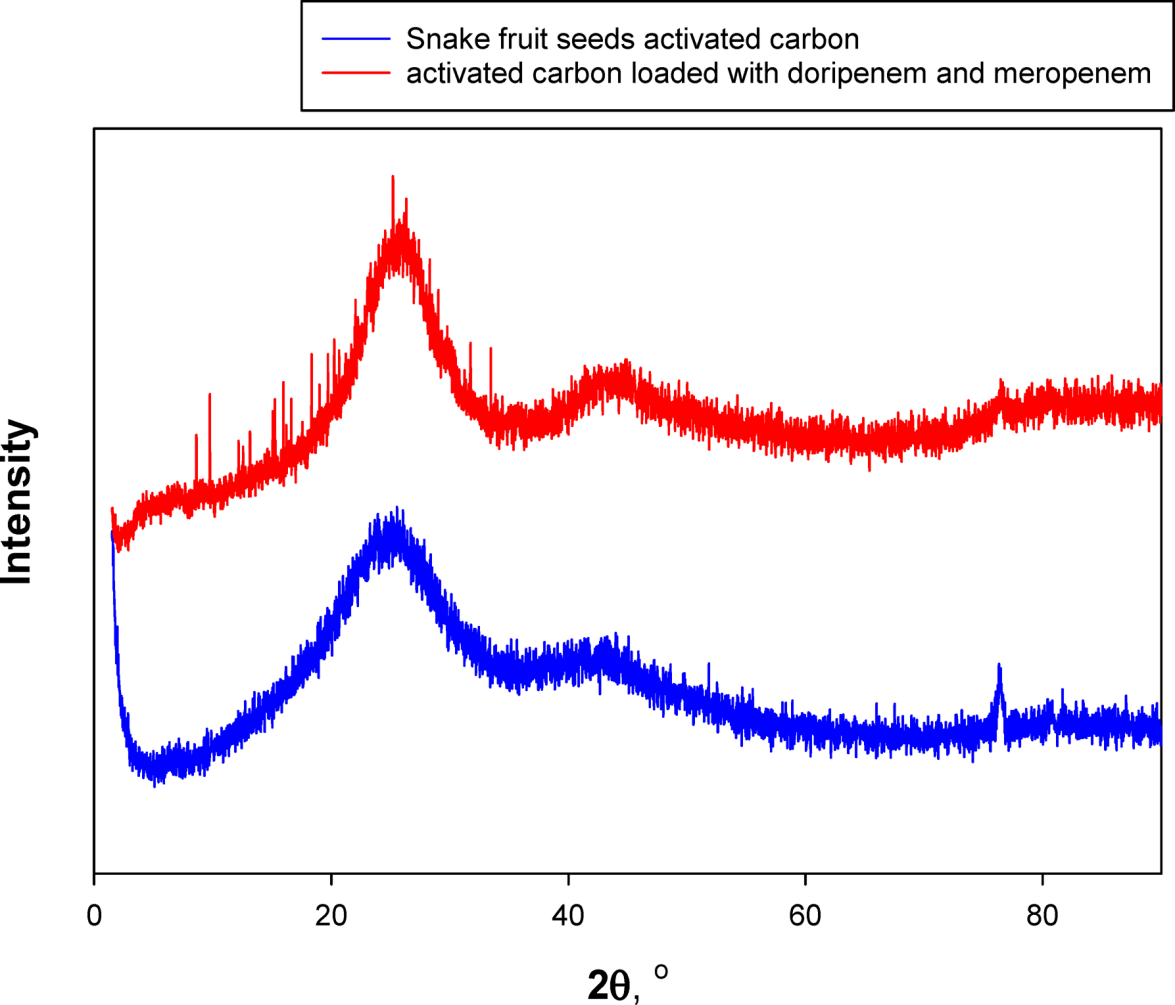
XRD spectra of activated carbon produced from snake fruit seed before and after adsorption of doripenem and meropenem.

Figure 2 shows the XRD spectrum obtained for snake fruit seed-derived AC (blue curve). The broad diffraction peak located around 2θ = 23° is associated with the (002) diffraction plane. The observation of such diffraction peak clearly reflects the turbostratic carbon structure. Moreover, the absence of sharp diffraction peaks indicated that the material is principally amorphous with low crystallinity. The aforementioned result is typical of biomass-derived activated carbons. Furthermore, the appearance of a smaller peak around 43°, connected to the (100) diffraction plane, further supports the presence of a disordered carbon structure. Such structural characteristics are advantageous for adsorption applications, as they provide high surface area and a wide distribution of micro- and mesopores.

After the adsorption of DOR and MER, significant changes were observed in the XRD patterns (red spectra) of AC surface. An increase in the intensity of the peak around 23° was detected, suggesting enhanced local ordering or possible stacking of adsorbate molecules on the AC surface. This broad peak corresponds to the (002) reflection of turbostratic carbon, a typical feature of amorphous or partially graphitized activated carbon derived from lignocellulosic biomass^25^. More notably, new sharp peaks emerged in the 2θ range of 10–30°, which were absent in the pristine activated carbon spectrum. These peaks correspond to the characteristic diffraction patterns of pure DOR and MER, as described above, indicating that the antibiotic molecules were successfully adsorbed onto the AC surface and retained part of their crystalline structure.

Peak position analysis confirmed that several characteristic peaks of DOR (e.g., 10.95°, 13.12°, 15.03°, 16.64°, 23.40°, and 26.13°) and MER (e.g., 8.62°, 12.56°, 15.23°, 19.79°, 22.03°, 24.54°, and 26.31°) also appeared in the spectra of the AC after adsorption. This provides a strong evidence that both antibiotics were simultaneously adsorbed, forming a crystalline deposit or thin molecular layer on the AC surface. The adsorption process is likely governed by multiple interactions, including hydrogen bonding, π–π stacking interactions between the aromatic moieties of the antibiotics and the carbon framework, as well as physical adsorption and possible chemical interactions.

The most significant change evident in Fig. 2 is the emergence of new crystalline peaks in the 10–30° range, which were not present in the original activated carbon. This confirms that the antibiotic structure remains detectable on the surface and that the adsorption process is occurring effectively.

The unchanged position of the main diffraction peak of activated carbon indicates that the fundamental carbon structure remains stable even after the adsorption process. This finding suggests that activated carbon derived from snake fruit seeds is not only effective in adsorbing DOR and MER molecules but is also structurally stable, maintaining its integrity following adsorption. The appearance of crystalline peaks corresponding to the antibiotics in the spectra further indicates that part of the adsorbed drug molecules are retained in a crystalline or semi-crystalline state, rather than being completely dissolved or degraded.

**Fig. 3:**
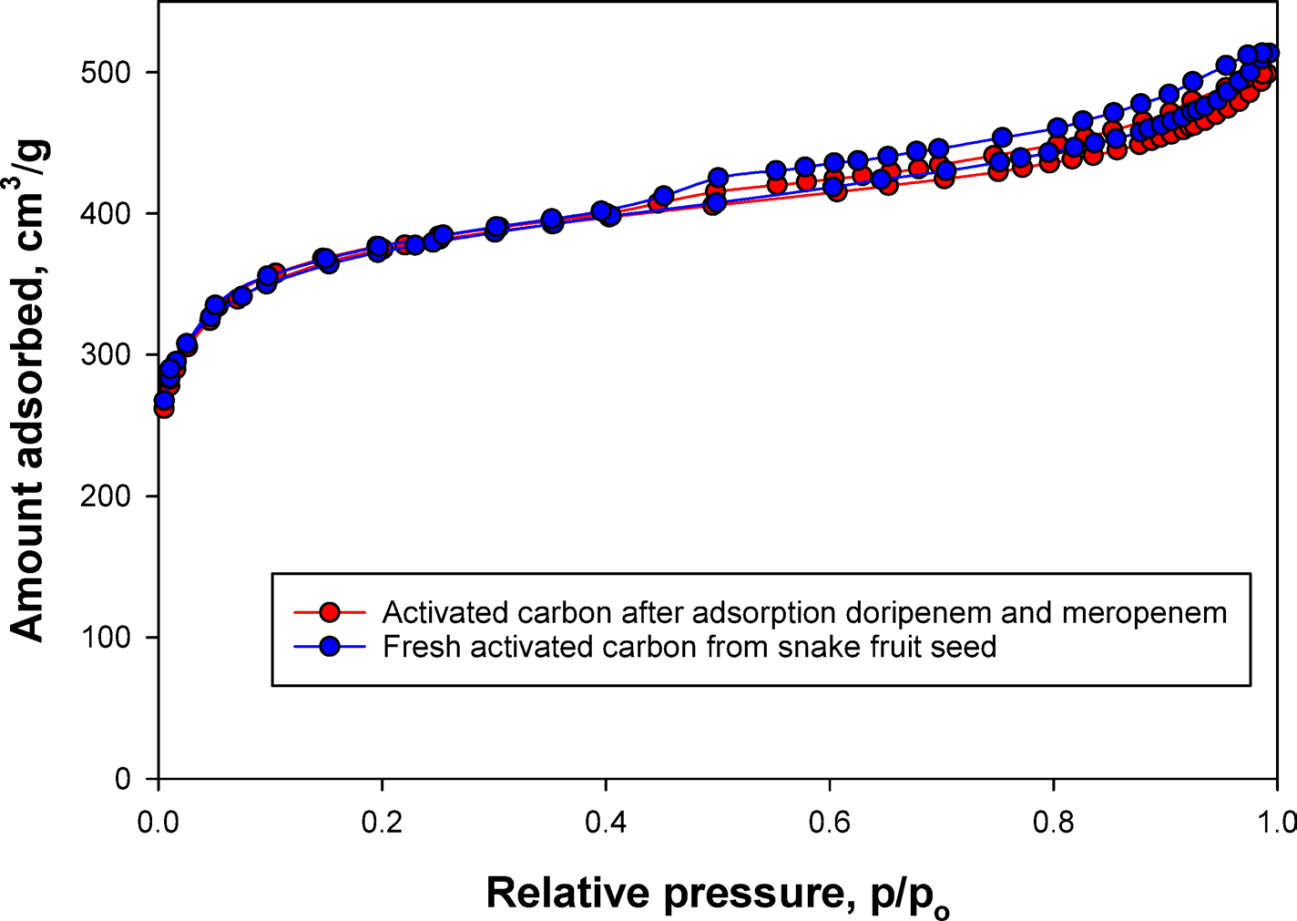
Nitrogen sorption isotherms of the activated carbon produced from snake fruit seed before and after adsorption of doripenem and meropenem.

The nitrogen adsorption–desorption isotherms (Fig. 3) compare the textural properties of AC derived from snake fruit seeds before and after antibiotic adsorption. The blue curve corresponds to fresh activated carbon, whereas the red curve represents the material after exposure to DOR and MER. Both samples exhibit type IV isotherms, according to the IUPAC classification, which are characteristic of mesoporous materials. These isotherms display a pronounced hysteresis loop at relatively high relative pressures (p/p₀ > 0.4), indicating the presence of a well-developed mesoporous network. Such structural features confirm the suitability of the material as an efficient adsorbent.

Quantitatively, the volume of nitrogen adsorbed on fresh AC was slightly higher than that on the antibiotic-exposed material, particularly at medium to high relative pressures. This reduction suggests that, following adsorption, some of the pores in the AC were partially blocked or filled with antibiotic molecules, thereby limiting nitrogen accessibility during the isotherm measurement. However, the decrease was modest, indicating that the pore structure of the AC remained largely open and did not undergo significant structural damage during adsorption.

As described in previous section "Activated carbon preparation", the activation process in this study consisted of two stages: KOH impregnation and physical activation using CO₂ gas. Chemical activation with KOH plays a role in forming the initial micropores through intercalation reactions and gas evolution. However, the subsequent activation stage using CO₂ at high temperatures acts as a mild oxidizing agent capable of widening the micropores through a partial gasification reaction (C + CO₂ → 2CO).This combination mechanism produces a carbon structure with dominant mesoporous properties, as indicated by the type IV isotherm curve, a clear hysteresis loop, and an average pore diameter in the mesopore range. Thus, the obtained mesoporous character is consistent with the two-step KOH–CO₂ activation technique.

BET surface area analysis supported these observations, showing a reduction from 1260.61 m^2^ /g for fresh activated carbon to 1221.38 m^2^/g after antibiotic adsorption. This corresponds to a decrease of approximately 3.1%, confirming that although DOR and MER were adsorbed, they did not completely block the pore system. Instead, adsorption likely occurred predominantly on the internal surfaces and pore walls, without causing collapse or substantial degradation of the porous network.

In addition to surface area, nitrogen adsorption–desorption analysis also provides information on the total pore volume and average pore diameter. Before adsorption, activated carbon from snake fruit seeds exhibits a total pore volume of 0.487 cm^3^/g with an average pore diameter of approximately 3.2 nm, which is consistent with the mesoporous character indicated by the type IV hysteresis in Fig. 3. After DOR and MER adsorption, the total pore volume slightly decreases to 0.473 cm^3^/g, while the average pore diameter changes to 3.1 nm. This decrease is consistent with a slight shift in nitrogen adsorption capacity at medium to high relative pressures. It confirms the occurrence of partial pore filling and pore blocking by antibiotic molecules, without causing significant structural change to the pore network.

The combination of a high specific surface area (1260.61 m^2^/g), a large total pore volume, and pore diameters in the mesoporous range is highly favorable for the adsorption of DOR and MER. Mesopores provide sufficiently wide diffusion channels to facilitate the transport of antibiotic molecules into the carbon structure, while the contribution of micropores and internal pore walls provides a large contact area for physical interactions such as van der Waals forces and π–π stacking with the carbon framework.

**Fig. 4:**
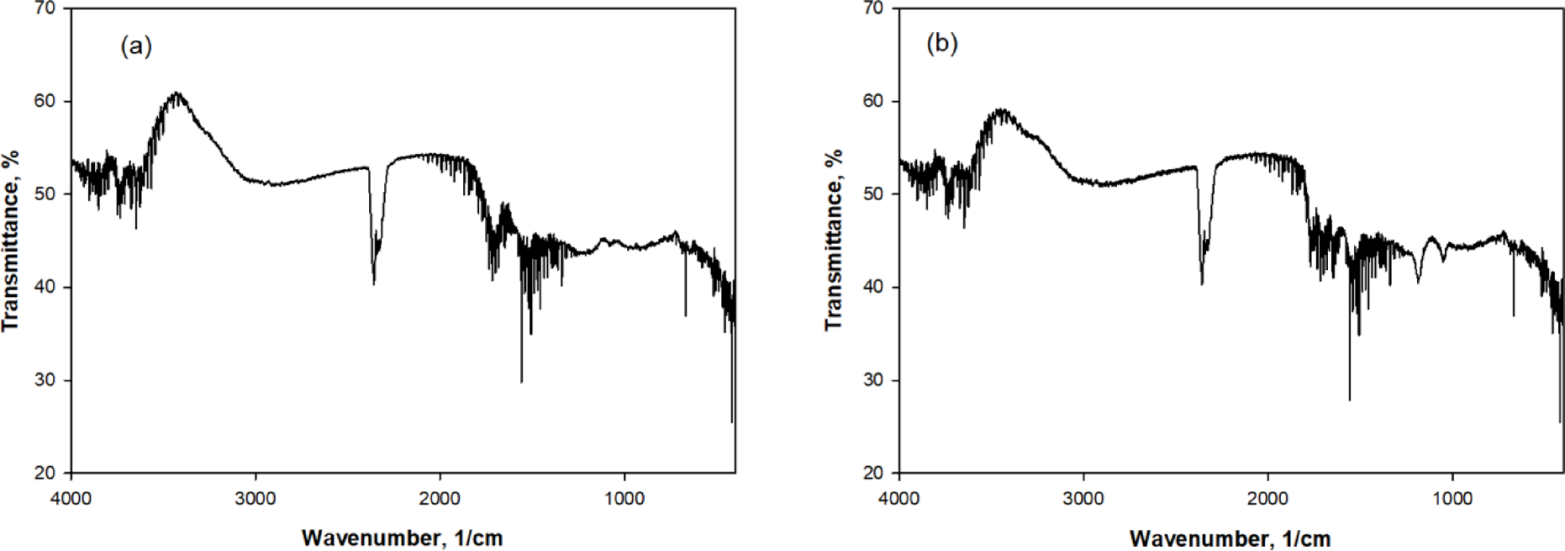
FTIR spectra of (**a**) activated carbon, and (**b**) activated carbon after adsorption of DOR and MER.

Figure 4 presents the FTIR spectra of (a) activated carbon and (b) activated carbon after adsorption of doripenem (DOR) and meropenem (MER). Prior to adsorption, the activated carbon exhibits a broad band centered at approximately 3400 cm⁻^1^, which is attributed to O–H stretching vibrations of surface hydroxyl groups and adsorbed moisture. These oxygen‐containing functionalities are commonly reported as active adsorption sites due to their strong hydrogen‐bond donor and acceptor characteristics. After adsorption of DOR and MER (Fig. 8b), this broad O–H/N–H stretching band becomes noticeably broadened and slightly attenuated in intensity, indicating the involvement of surface hydroxyl groups in hydrogen bonding interactions with polar functional groups of the antibiotic molecules, particularly amide and β-lactam moieties. This observation suggests that hydrogen bonding plays a dominant role in anchoring DOR and MER onto the activated carbon surface.

The absorption band around 1700–1650 cm⁻^1^, assigned to C=O stretching vibrations of surface carbonyl groups on activated carbon, shows an increase in intensity and slight shape modification after adsorption. This region overlaps with the amide I (C=O stretching) vibrations characteristic of β-lactam antibiotics, indicating strong dipole–dipole interactions and possible hydrogen bonding between the carbonyl groups of the adsorbent and the amide functionalities of DOR and MER.

Additionally, the region between 1200 and 1000 cm⁻^1^, associated with C–O stretching vibrations of phenolic, alcoholic, and ether groups on activated carbon, exhibits enhanced absorption after adsorption. This change reflects the contribution of polar surface oxygen groups to adsorption through secondary interactions with C–N, C–O, and sulfonyl‐related functionalities present in the antibiotic molecules.

Importantly, no new absorption bands are observed after adsorption, indicating that the interaction between activated carbon and DOR/MER is predominantly physical in nature rather than involving covalent bond formation. The adsorption process is therefore governed by a combination of hydrogen bonding, polar interactions, and surface affinity between oxygen‐rich functional groups of activated carbon and heteroatom‐containing functional groups of the antibiotics.

Overall, the FTIR results confirm that surface hydroxyl, carbonyl, and ether‐type functional groups of activated carbon play a critical role in the adsorption of DOR and MER, supporting the proposed adsorption mechanism and complementing the adsorption performance results discussed elsewhere in the manuscript.

### Adsorption isotherms in single-compound and binary systems

**Fig. 5:**
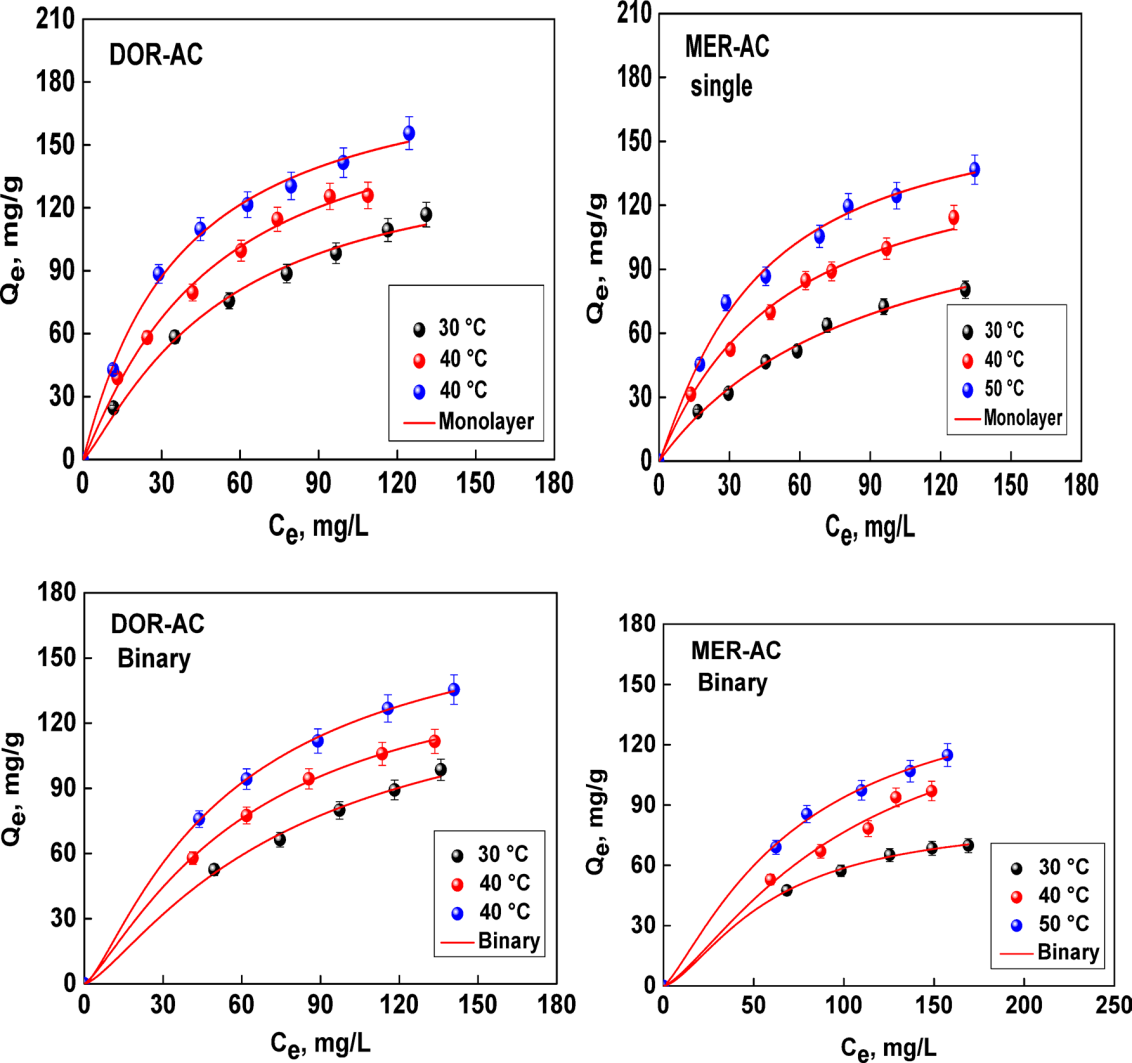
Single-compound and binary adsorption isotherms of doripenem and meropenem at different temperatures. Data fitting by statistical physic models.

Adsorption isotherms of both DOR and MER on the produced adsorbent were determined in single-compounds and binary solutions, as reported in Fig. 5.

Adsorption patterns follow a similar trend for all the experimental conditions investigated. In both single-compound and binary systems, the AC shows a slightly higher adsorption capacity towards DOR molecules, and the differences increase along with temperature. In all the systems investigated, the temperature plays a positive role, as adsorption capacities increase with an increase in temperature, regardless of the considered adsorbate. This result could be likely ascribed to a decrease in water adsorption, which can exert competition on adsorbates adsorption^26,27^. Finally, experimental evidences highlight that for both DOR and MER a competitive effect in binary system occurs, which is more significant for MER molecules, i.e. the adsorbate that shows a lower adsorption capacity (and, then, affinity) in single-compound systems. This result is consistent with many other adsorption systems, whose results are available in the pertinent literature, also dealing with different adsorbates^12,28–30^.

Molecularly, DOR and MER have different sizes and polarities, which directly contribute to their competitive adsorption behavior on the AC surface of snake fruit seeds. DOR has a molecular mass of approximately 420 g/mol, a topological polar surface area (TPSA) of ~ 196 Å^2^, and an XLogP value of ≈ –4.2, while MER has a molecular mass of approximately 383 g/ mol, a TPSA of ~ 135 Å^2^, and an XLogP value of ≈ –0.8. These TPSA and XLogP values indicate that DOR is larger and much more polar than MER, with a higher number of hydrogen bond donor and acceptor groups. This is in line with their chemical structures: DOR carries sulfonamide groups and more heteroatom functionalities (O, N, S), while MER has a relatively less polar dimethyl carbamoyl side chain.

These differences in physicochemical properties correlate with the single and binary adsorption data. In the single system, the maximum capacity indicates that AC has a slightly higher affinity for DOR. However, the competitive effect became more pronounced when both antibiotics were present simultaneously. For instance, at 30 °C, the MER capacity decreased from single system binary system, while the DOR capacity under the same conditions remained virtually unchanged. This pattern indicates that DOR has stronger affinity and higher competition for activated carbon receptor sites (ACRS), as the presence of DOR more easily inhibits MER adsorption.

From the perspective of molecule–surface interactions, the combination of slightly larger molecular size, higher polarity, and a greater number of hydrogen bond donors/acceptors allows DOR to form a stronger interaction network with oxygen functional groups (e.g., –OH, C–O, and C=O) on the surface of snake fruit AC. Highly polar DOR also carries a strong hydration layer, but once it successfully enters the mesopores and approaches the active site, its numerous polar groups and zwitterionic charges enable the formation of multipoint hydrogen bonds and local electrostatic interactions, resulting in a higher effective adsorption energy per molecule compared to MER. Conversely, slightly smaller and less polar MER (lower TPSA and |XLogP|) has a more limited hydrogen bonding ability with the AC surface, making it more easily eliminated when directly competing with DOR for the same site.

It should be noted that the effect of pH was not systematically varied in this study. All adsorption experiments were performed at the native pH of the working DOR and MER solutions, which is near neutral. Based on the reported pKa values, both antibiotics predominantly exist as zwitterionic species in this pH region, resulting in minimal net charge. Under these conditions, electrostatic forces contribute less to adsorption, while hydrophobic interactions, π–π interactions with the carbon skeleton, dipole–dipole interactions, and hydrogen bonding are expected to dominate the adsorption mechanism. These fixed pH conditions provide a consistent basis for evaluating both single and competitive adsorption behavior; however, we acknowledge that systematic pH-dependent studies could further clarify how pH-driven molecular speciation affects adsorption energetics. This represents a crucial direction for future research.

Finally, in Table 1, a comparison of the performances of similar adsorbents for the adsorption of antibiotics from contaminated water is reported:

**Table 1:** Maximum β-lactam antibiotics adsorption capacities (Q_m_) of different adsorbents reported in the literature.

Systems	Q_m_(mg/g)	References
DOR-activated carbon	193.37	This study
DOR-CNC	65.7	^31^
DOR-CNC-alginate	98.4	^31^
DOR- organobentonite	59.606	^32^
MER- activated carbon	171.06	This study
MER- biochar	80	^33^
MER-forest biomass	17.2	^34^
MER-CFO@Au nanomaterials	25.5	^35^
MER-Fe3O4-MER-MMIPs	11.49	^36^

The reported data clearly highlights the competitive level of the performances retrieved for the adsorbent produced in the present studies when compared with other samples available in the pertinent literature.

### Modelling analysis by statistical physic models

The adsorption mechanisms of DOR and MER on the investigated AC in both single-compound and binary systems were analyzed through a modelling analysis adopting the models presented in Section "Single-compound and competitive adsorption models". In Fig. 5, the model fittings are reported, testifying a good capacity of the selected models in interpreting the experimental evidences. The fitting of the experimental data allowed determining the corresponding parameter values at different temperatures, which can be further analyzed to retrieve interesting properties and behavior of the adsorption systems under investigation. The results are presented in Tables 2 and 3.

**Table 2:** Fitting parameters of the model for single-compound systems.

T, °C	R^2^	n	Δn	N_m_, mg/g	ΔN_m_, mg/g	C_1/2_, mg/L	Q_s_, mg/g	Δ Q_s_, mg/g	ΔE ,kJ/mol
DOR-AC									
30	0.993	1.21	0.060	122.48	6,124	51.65	148.20	7,410	11.51
40	0.995	1.11	0.055	161.48	8,074	47.31	179.24	8,962	12.12
50	0.996	1.04	0.052	185.94	9,297	36.40	193.37	9,668	13.19
MER-AC									
30	0.992	0.99	0.049	139.59	6,9795	91.76	138.19	6,909	11.81
40	0.995	1.03	0.051	148.68	7,434	52.82	153.14	7,657	13.64
50	0.993	1.12	0.056	152.74	7,637	41.21	171.06	8,553	14.74

**Table 3:** Fitting parameters of the model for binary systems.

T, °C	R^2^	n_i (i=1,2)_	Δn_i_	N_m_, mg/g	Δ N_m_, mg/g	C_01,02_, mg/L	Q_si_, mg/g	Δ Q_si_, mg/g	ΔE ,kJ/mol
DOR-AC									
30	0.995	1.03	0,051	145.23	7,261	57.96	149.35	7.4675	11.22
40	0.999	1.25	0,062	124.25	6,212	34.68	155.31	7.7655	12.93
50	0.999	1.69	0,084	108.20	5,410	24.25	182.85	9.1425	14.31
MER-AC									
30	0.998	1.52	0,076	55.19	2,759	37.04	83.88	4.194	14.10
40	0.986	1.36	0,068	110.40	5,520	98.23	150.14	7.507	12.03
50	0.998	1.27	0,063	122.77	6,138	72.88	155.91	7.795	13.21

The theoretical results indicate that the maximum adsorption capacities in single-compound systems are 148, 176, and 193 mg/g for DOR, and 138, 153, and 171 mg/g for MER at the three investigated temperatures. Importantly, the difference in single adsorption capacities is not significant, that indicates the adsorbent has a similar affinity to detect both pollutants (MER and DOR). This comparison showed the importance of the adsorbent used in this work to remove various pharmaceuticals and can be a relevant material for industrial applications. The binary adsorption capacities are: 149, 155, and 182 mg/g for DOR, and 83, 150, and 155 mg/g for MER. The presence of a second adsorbate in solution reduced the adsorbent performance, particularly for MER.

Overall, the results confirm that the presence of one antibiotic inhibits the adsorption of the other, a behavior that has also been reported for other pharmaceutical contaminants in competitive adsorption systems (e.g.^12^).

**Fig. 6:**
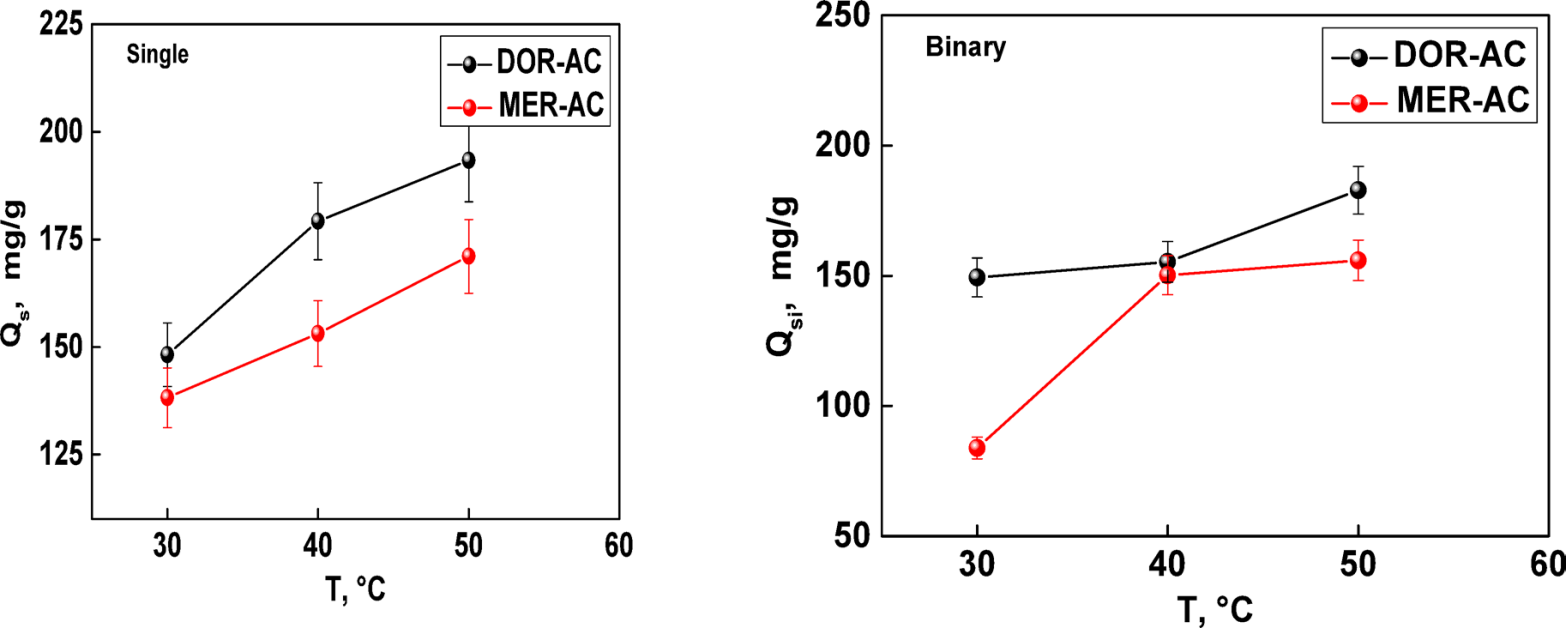
Maximum adsorption capacities in single-compound and binary systems as a function of temperature.

Furthermore, the influence of temperature was investigated to better understand the adsorption mechanism (Fig. 6). It was observed that adsorption capacities increased with rising temperature, confirming that the process is endothermic. Comparable results were retrieved in similar applications (e.g.^29^), while opposite behaviors are more frequent (e.g.^37^). Moreover, this finding is particularly relevant for practical applications, as higher temperatures appear to enhance the overall adsorption performance of AC.

**Fig. 7:**
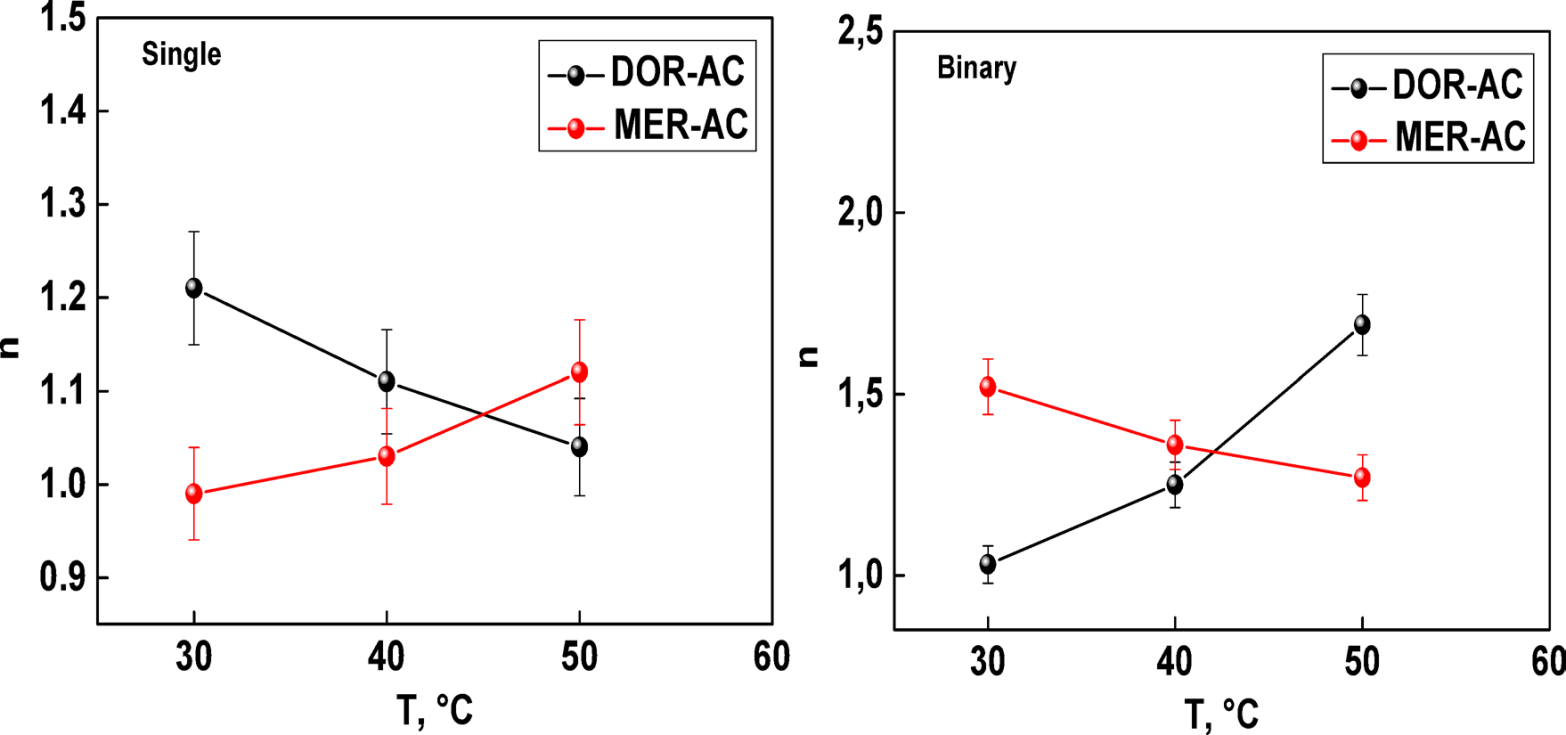
Number of DOR and MER molecules bound per AC active site in single-compound and binary systems.

In both the models, more than one parameter can be used to describe the competitive effects observed in the single-compound and binary adsorption data. Besides adsorption capacities, the number of DOR and MER molecules detected per AC active site also helps to define the adsorption mechanisms. The effect of temperature on this parameter is illustrated in Fig. 7. As assumed in the binary adsorption model, each AC active site can adsorb either DOR or MER molecules. The results show that the number of molecules per site varied in opposite directions, indicating that when one contaminant interacts with an AC, the other is excluded. This behavior confirms the antagonistic effect of competition for the same receptor sites. Overall, it can be deduced that both contaminants share the same AC active site.

In single-component systems, the number of DOR and MER molecules bound per AC active site was lower across most temperatures. This indicates that both adsorbates were adsorbed individually without aggregation, due to weaker intermolecular interactions (MER–MER or DOR–DOR) prior to adsorption.

**Fig. 8:**
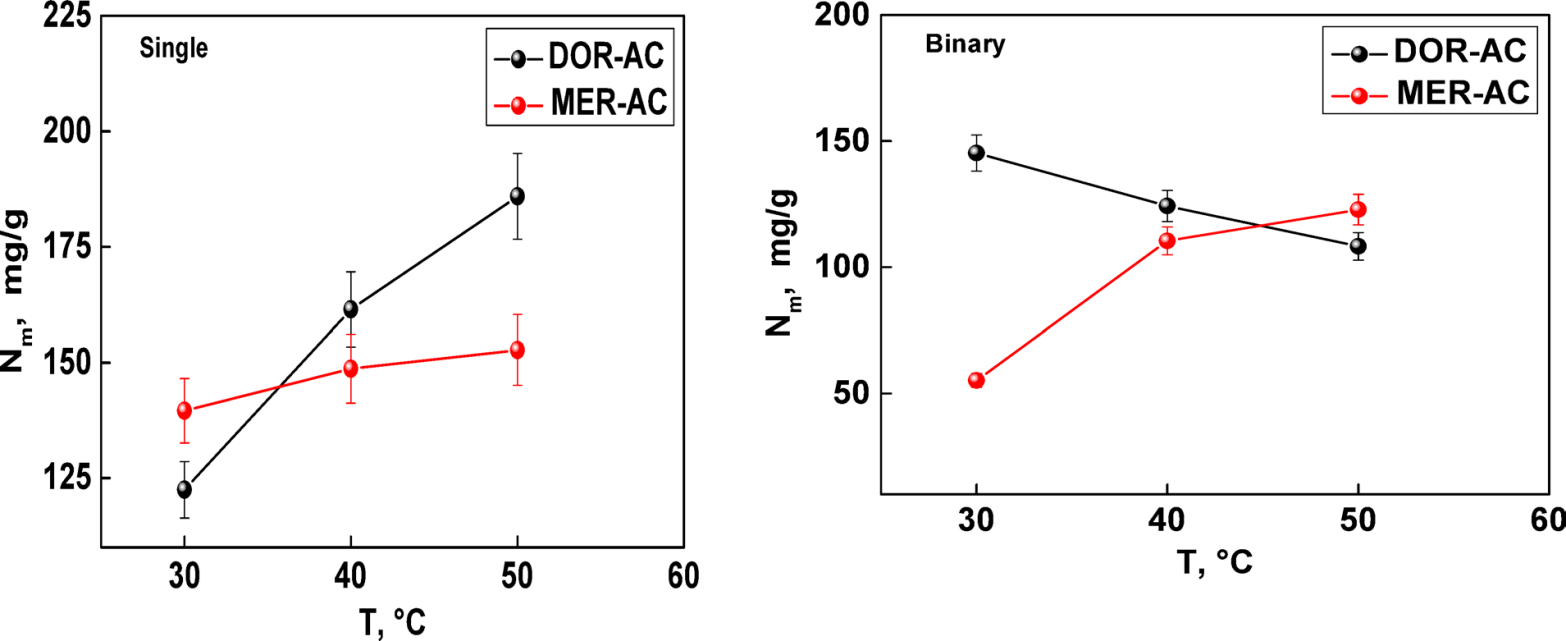
Adsorption densities of DOR and MER molecules in single-compound and binary systemsas function of temperature.

Figure 8 describes the dependence between temperature and densities of receptor sites in single and binary systems. For single systems, adsorption density increased with temperature, indicating that higher thermal energy promotes adsorption, as previously already observed. This effect is likely due to a decrease in water adsorption by raising temperature, which may generate additional accessible ACRSs for adsorbates removal. In contrast, binary adsorption densities evolved in opposite directions, reflecting the strong competitive effect between DOR and MER molecules.

Finally, to further elucidate the adsorption mechanism, the adsorption energies of all systems were determined by fitting the single and binary adsorption data using the following relationships:

4 $$\Delta E_{1,2} = RT\ln \left( {\frac{{C_{s} }}{{C_{1,2} }}} \right)$$

5 $$\Delta E_{1,2} = RT\ln \left( {\frac{{C_{s} }}{{C_{01,02} }}} \right)$$

where Cs is the adsorbate solubility in water and R is the ideal gas constant. The calculated values are reported in Tables 2 and 3. All adsorption energies were positive and relatively low, confirming that both single and binary adsorption processes are endothermic and governed by physisorption.

Based on the modeling results (Tables 2 and 3), the maximum adsorption capacity (Qs) for DOR and MER increased consistently when the temperature was increased from 30 to 50 °C, and all adsorption energies obtained were positive but relatively small, indicating an endothermic adsorption process dominated by physisorption. The increase in capacity with increasing temperature can be explained by several factors: (i) hydrodynamically, increasing temperature decreases the viscosity of water and increases the molecular diffusion coefficient (D ∝ T/η), thereby accelerating external diffusion through the liquid film and facilitating intra-particle diffusion within the AC mesoporous network; (ii) thermodynamically, increasing temperature strengthens the entropy contribution (ΔS > 0) due to the release of structured water molecules from the carbon surface and from the hydration shell of DOR/MER, so that the − TΔS term becomes more dominant and makes the adsorption ΔG more negative even though ΔH is positive; and (iii) moderate changes in the surface energy and ionization state of oxygen groups on the AC surface can also slightly modify the attractive forces between the carbon surface and the polar/zwitterionic groups of DOR–MER, but their contribution remains consistent with the physisorption mechanism supported by the low adsorption energy values.

### Adsorbate–adsorbent interaction mechanism

**Fig. 9:**
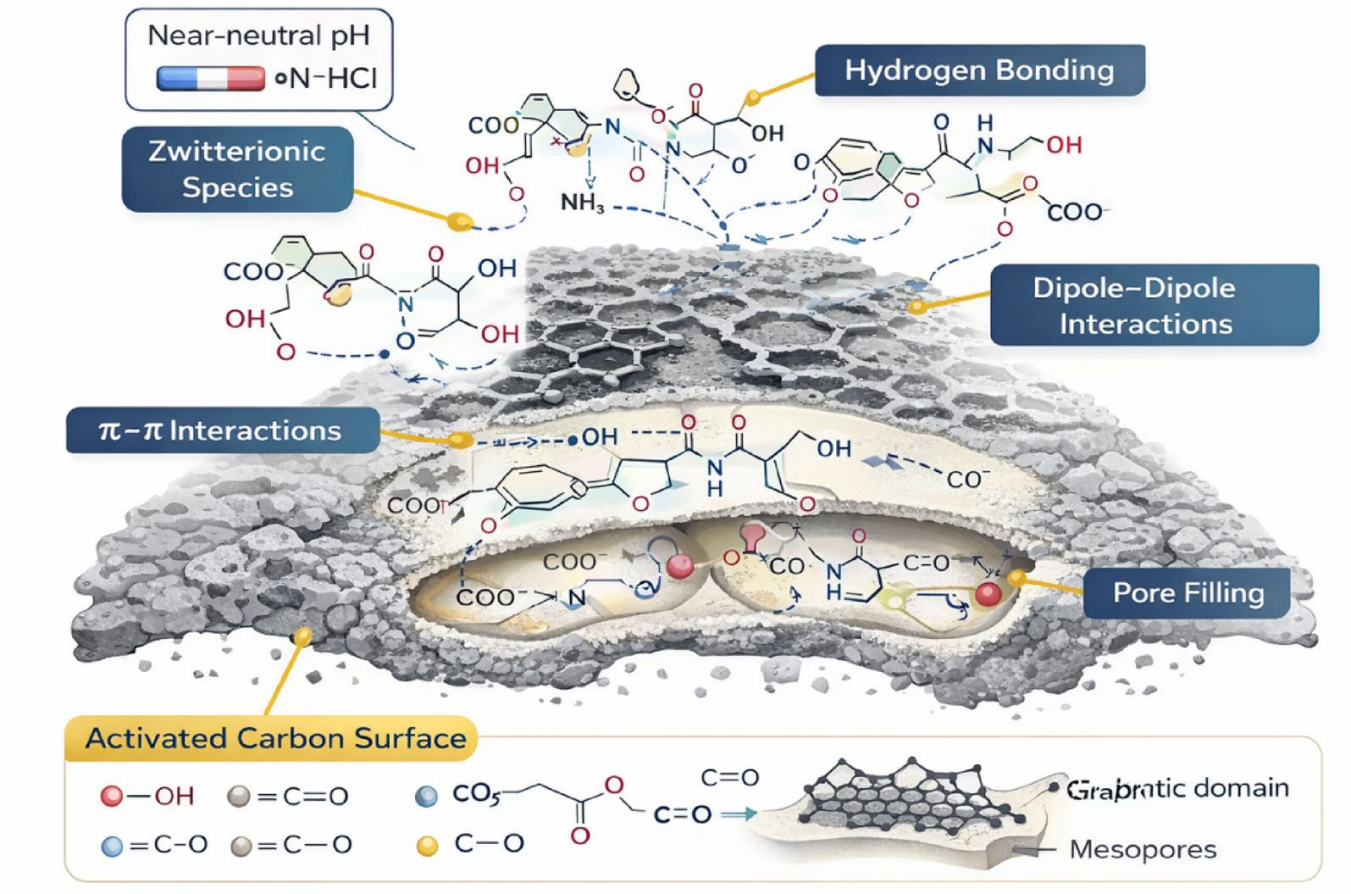
Adsorbent-adsorbate interaction mechanism.

The adsorption mechanism of doripenem (DOR) and meropenem (MER) on activated carbon derived from snake fruit seeds is governed by a combination of surface chemistry, pore structure, and solution conditions. Under the natural pH of the antibiotic solutions (near neutral), both DOR and MER predominantly exist in zwitterionic form, minimizing electrostatic interactions. Consequently, adsorption is mainly driven by physical–chemical interactions rather than ionic attraction (Fig. 9).

Oxygen-containing functional groups on the activated carbon surface, such as hydroxyl (–OH), carbonyl (C=O), and ether (C–O) groups, play a crucial role by forming hydrogen bonds and dipole–dipole interactions with polar functional moieties of DOR and MER, including amide, β-lactam, and sulfonamide groups. In addition, weak π–π interactions may occur between the heterocyclic rings of the antibiotics and the graphitic domains of the carbon surface.

The mesoporous structure enhances molecular diffusion and pore filling that allows efficient access to internal adsorption sites (Fig. 9).

The absence of new FTIR bands after adsorption, together with the low adsorption energies derived from statistical physics modeling, confirms that the process is dominated by reversible physisorption rather than chemisorption. This mechanistic interpretation is consistent with the observed adsorption behavior in both single-compound and binary systems.

### Regeneration and reusability of the adsorbent

**Fig. 10:**
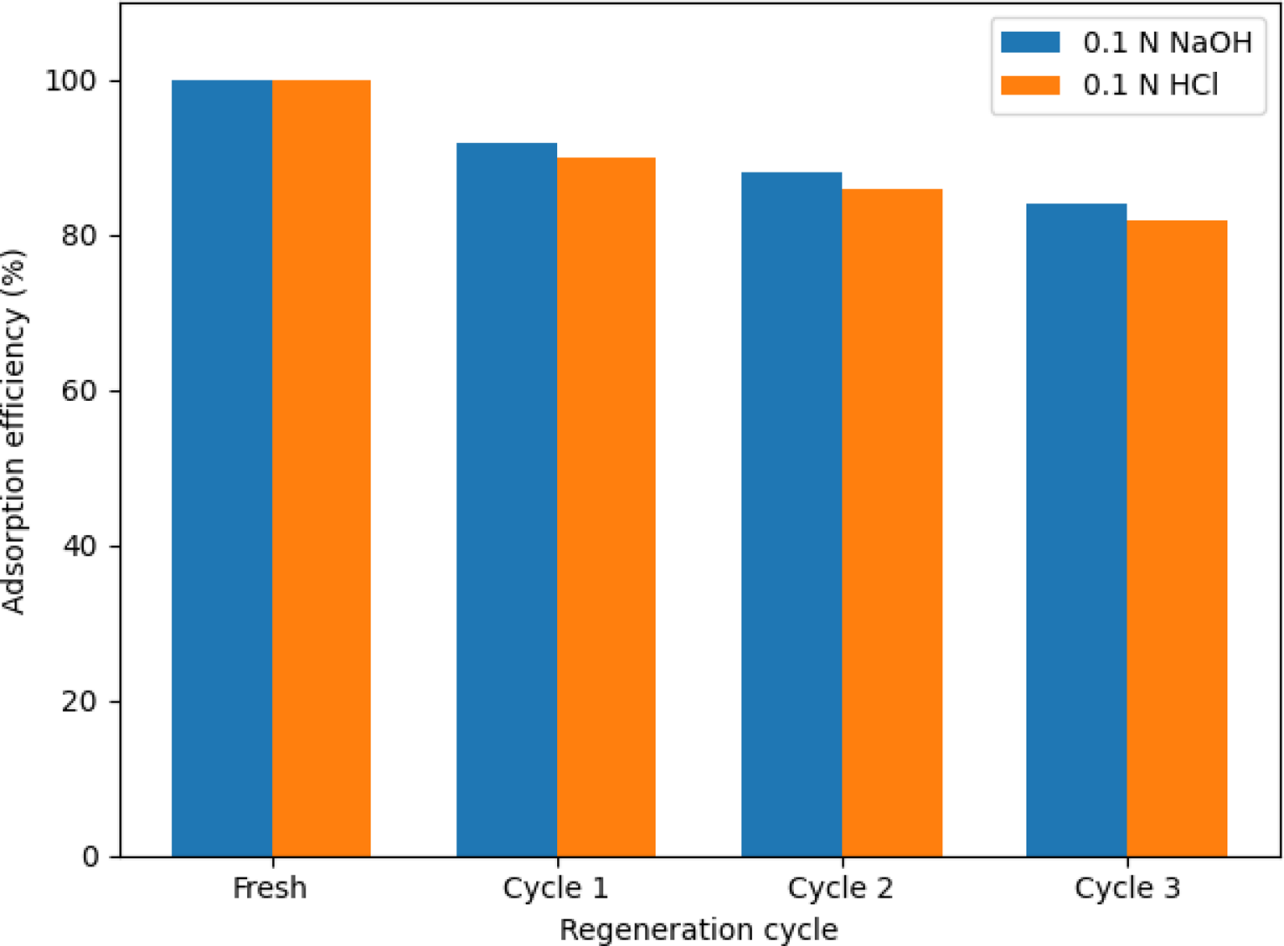
Adsorption efficiency of activated carbon over successive regeneration cycles using 0.1 N NaOH and 0.1 N HCl as eluting agents.

The regeneration performance of the adsorbent was evaluated using chemical desorption with 0.1 N NaOH and 0.1 N HCl solutions. As shown in Fig. 10, the fresh adsorbent exhibited 100% adsorption efficiency, which gradually decreased after repeated adsorption–desorption cycles. After three regeneration cycles, the adsorption efficiency remained above ~ 80% for both eluting agents, indicating good structural stability and reusability of the adsorbent. A slightly higher regeneration efficiency was observed with NaOH as the eluent compared to HCl. This behavior can be attributed to the ability of alkaline solutions to more effectively disrupt hydrogen bonding and polar interactions between the antibiotic molecules and oxygen-containing functional groups on the activated carbon surface. In contrast, acidic regeneration may be less effective at desorbing firmly bound polar moieties, resulting in a marginally higher capacity loss. Overall, the relatively high adsorption efficiency retained after multiple regeneration cycles confirms that reversible physisorption mechanisms predominantly govern the adsorption of doripenem and meropenem. The mild decrease in performance is likely associated with partial pore blockage or incomplete desorption of a fraction of the adsorbed molecules, rather than structural degradation of the activated carbon.

## Future perspective

This research indicates that our adsorbent (snake fruit seed-based activated carbon) played a relevant role for removing pharmaceuticals such as MER and DOR. In this direction, additional research is required to ensure its applicability in different industrial sectors. First, the regeneration and reusability of the adsorbent need to be evaluated through both thermal and chemical desorption cycles to assess its durability in long-term operation. Second, further studies using continuous-flow column systems are needed to obtain information on breakthrough behavior and dynamic adsorption capabilities under operating conditions more closely to field applications. Third, testing in real wastewater or other complex matrices containing natural organic compounds, inorganic ions, and various pharmaceutical contaminants is needed to understand the selectivity of the adsorbent and the influence of interferents on its performance. In conclusion, these approaches are important for advancing the development of snake fruit seed-based biosorbents for more real applications in water treatment.

## Conclusions

In this work, we investigated single and binary adsorption of two β-lactam antibiotics on activated carbon prepared from snake fruit seeds was investigated. By combining experimental results with advanced theoretical models, the adsorption mechanisms were elucidated and discussed. Experimental results showed that the activated carbon had high performance for both antibiotics in single systems, reaching up to 193 mg/g for DOR and 171 mg/g for MER, with only minor differences between the two. This result designates that the adsorbent possesses a comparable affinity for both pollutants. In binary systems, however, adsorption capacities decreased significantly, highlighting a strong competitive effect when both antibiotics were present simultaneously. Advanced theoretical models were applied to explain the adsorption of these pharmaceuticals on the investigated adsorbent. The analysis of model parameters such as the number of DOR and MER molecules per site showed that they varied in opposite manner, signaling that when one pharmaceutical interacts with an AC, the other is excluded. The modelling investigation indicated also that the presence of a second adsorbate in solution created an antagonist effect between DOR and MER on the same adsorbent site via the estimation of adsorption capacity ratios. The calculation of adsorption energies corroborated that the adsorption systems are endothermic and based on physisorption. Overall, this work demonstrates that snake fruit seed–derived activated carbon is an efficient, stable, and sustainable adsorbent for removing emerging pharmaceutical contaminants from aqueous solutions.

## Data Availability

The data that supports the findings of this study are available from Lotfi Sellaoui, but restrictions apply to the availability of these data, which were used under license for the current study, and so are not publicly available. Data are, however, available from the corresponding author upon reasonable request and with permission of Lotfi Sellaoui.
